# How do labour market conditions explain the development of mental health over the life-course? A conceptual integration of the ecological model with life-course epidemiology in an integrative review of results from the Northern Swedish Cohort

**DOI:** 10.1186/s12889-024-18461-6

**Published:** 2024-05-15

**Authors:** Anne Hammarström, Hugo Westerlund, Urban Janlert, Pekka Virtanen, Shirin Ziaei, Per-Olof Östergren

**Affiliations:** 1 Unit of Occupational Medicine, Institute of Environmental Medicine, Karolinska Institutet, Solnavägen 4, Stockholm, 113 65 Sweden; 2 Department of Epidemiology and Global Health, Umeå University, Umeå, Sweden; 3 Stress Research Institute, Stockholm University, Stockholm, Sweden; 4 Faculty of Social Sciences, Tampere University, Tampere, Finland; 5 Social Medicine and Global Health, Department of Clinical Sciences in Malmö, Lund University, Lund, Sweden

**Keywords:** Theories, Labour market, Mental health, Ecosocial theory, Life-course theories, Embodiment, Agency, Societal efforts

## Abstract

**Background:**

The aim of this study was to contribute to the theoretical development within the field of labour market effects on mental health during life by integrating Bronfenbrenner’s ecological model with mainly earlier theoretical work on life-course theory.

**Methods:**

An integrative review was performed of all 52 publications about labour market conditions in relation to mental health from the longitudinal Northern Swedish Cohort study. Inductive and deductive qualitative content analysis were performed in relation to Bronfenbrenner’s ecological framework combined with life-course theories.

**Results:**

The following nine themes were identified: 1. Macroeconomic recession impairs mental health among young people. 2. The mental health effects on individuals of youth unemployment seem rather insensitive to recession. 3. Small but consistent negative effect of neighbourhood unemployment and other work-related disadvantaged on individuals’ mental health over life. 4. Youth unemployment becomes embodied as scars of mental ill-health over life. 5. Weak labour market attachment impairs mental health over life. 6. Bidirectional relations between health and weak labour market attachment over life. 7. Macrolevel structures are of importance for how labour market position cause poor health. 8. Unequal gender relations at work impacts negatively on mental health. 9. The agency to improve health over life in dyadic relations. Unemployment in society permeates from the macrolevel into the exolevel, defined by Bronfenbrenner as for example the labour market of parents or partners or the neighbourhood into the settings closest to the individual (the micro- and mesolevel) and affects the relations between the work, family, and leisure spheres of the individual. Neighbourhood unemployment leads to poor health among those who live there, independent of their employment status. Individuals’ exposure to unemployment and temporary employment leads to poorer mental health over the life-course. Temporal dimensions were identified and combined with Bronfenbrenner levels into a contextual life-course model

**Conclusion:**

Combining the ecosocial theory with life-course theories provides a framework for understanding the embodiment of work-related mental health over life. The labour market conditions surrounding the individual are of crucial importance for the embodiment of mental health over life, at the same time as individual agency can be health promoting. Mental health can be improved by societal efforts in regulations of the labour market.

## Background

An integrated understanding of how work-related factors influence mental health over life is lacking. In this integrative review we perform qualitative analyses of publications about the labour market effects on mental health during life and develop a contextualised life-course model about the embodiment of mental health during life.

To get a more holistic understanding of the impact of working conditions on mental health during life, this study will integrate main theoretical frameworks like the life-course model [1] with the theory of embodiment [2] and the ecosocial theory [2] in order to underline the concrete interplay between body and environment over the life-course of an individual. Urie Bronfenbrenner [3], stands out as one of the most influential contributors to ecological thinking in mental health research [2]. We suggest that Bronfenbrenner’s Ecological Systems Theory could serve as device for integrating a life course approach with the theory of embodiment, since it provides a multilevel systemic representation of exposures which relate to the axis of time for the individual.

Meta-analyses and systematic reviews have demonstrated that single work-related factors, such as lack of decision latitude, job strain and bullying, increase the risk of depressive symptoms [4]. The work-stress research has mainly focused on single measurements during life, and therefore loose the effect of intraindividual changes of working and employment conditions over time [5]. A life-course perspective is needed to capture exposures to various positions on the labour market during working life. During recent decades, the labour market has gone through rapid changes with increased insecurity for many workers. Globalization has led to new patterns of production, which have changed the working-life world-wide. These changes include increased polarisation between high- and low-paid jobs in relation to e.g., income. The demand for a more flexible workforce has led to a variety of non-standard work arrangements (e.g., short-term contracts, on call jobs, ‘platform gigs’, etc.) with increased levels of unemployment and widespread job insecurity as a result [6]. Employees in high-skill jobs have achieved increased autonomy and expanded their opportunities, while those in low-skill jobs have faced an increasingly insecure work situation with poorer employment relations and working conditions. During the last two decades, the number of fixed-term contracts has almost doubled among short time educated in Sweden [7]. So, while previous generations have worked with quite stable labour market position during life, those in working age of today have been exposed to rapid changes with increased risk for frequent transitions between various types of employment contracts, or jobs without a contract, and periods of unemployment, labour market measures or studies in-between. Therefore, a more holistic understanding is needed of the complex relations during life between health and work -related exposures in terms of both processes of exposures and processes of health-related selection which may influence individual’s’ vulnerability to poor work conditions [8].

To increase the understanding of these complex relations, there is a need for theoretical development in the field. So far, some models have been developed to understand how single work-related exposures influence health. The two most influential models in public health research used to analyse the role of psychosocial work environment in explaining health development are the demand, control, support model [9] and the effort-reward imbalance model [10]. A few older theories try to explain the health consequences of unemployment. According to Marie Jahoda’s theoretical framework [11], unemployment impairs health through the loss of both manifest (income) and latent (time structures, social networks, social identity, self-realisation, activity, and participation in collective effort) functions of employment. In stress theory, psychosocial threats (such as precarious employment or unemployment) together with psychobiological programming (including effects of earlier environmental and genetic factors) elicit metabolic and mental health responses [12, 13]. Other aspects of the working life include labour market attachment which has been conceptualized as a centre periphery structure with those with most secure attachment in the centre, while those with the most unsecure attachment are found in the periphery [14]. The core-periphery model aims to capture this heterogeneity by regarding the permanent employees as the ‘core work force’ with favourable working conditions and temporary employees as the ‘periphery work force’ with increasing insecurity and reduced benefits the longer in the periphery they are positioned.

The social context surrounding an individual has been emphasised to be of particular importance for understanding the development of mental health [3]. The concept of embodiment and the ecosocial truth theory has been added by Krieger to underline the concrete interplay between body and environment over the life-course of an individual [2].

To get a more holistic understanding of the impact of working conditions on mental health during life, this study will integrate life-course theories with a social ecological model developed by Bronfenbrenner, which recognises the effect of multiple and inter-related settings on various contextual levels on mental health. An ecosocial perspective offers a way to simultaneously emphasise both the individual and the context and the relations between them. However, the ecosocial model does not extensively elaborate the complexity of differential exposure across different life periods. An understanding of how work-related factors influence mental health over life, which integrates main theoretical frameworks like the life course model [1] and the theory of embodiment [2] is lacking. In this integrative review we develop a contextualised interpretation of the findings utilizing these frameworks. Mental health will be defined as self-reported symptoms of mainly depressiveness, anxiety and functional somatic symptoms.

The life-course of an individual is shaped by historical times, places, and processes in which an individual is embedded during life. The theoretical ground in life-course research was laid by the sociologist Glen Elder [1] by defining the following four principles (1) *historical time and place* mean that the life-course of individuals is embedded in and shaped by the historical times and places they experience over life (2) *Timing* in lives states that the developmental impact of events is contingent on when they occur in a person’s life. (3) *Linked lives* means that lives are lived interdependently, and social and historical influences are expressed through this network of shared relationships. (4) *Agency* states that individuals construct their own life-course through the choices and actions they take within the opportunities and constraints of history and social circumstances. The principles also should direct the research in the domain of life-course epidemiology.

The contextual approach and mixed method design emphasised by Elder has however, gradually been replaced by a quite narrow and descriptive focus on individual characteristics in the field of life-course epidemiology of today. Life-course epidemiology investigates how exposures earlier in life can explain later development of diseases, with a focus on how biological, behavioural, and psychosocial processes operate across the life span [15]. The main conceptual models are accumulation of exposures, social chain of risks and critic or sensitive periods, implying increased sensitivity in a particular stage of the individual’s life when a certain exposure has the strongest detrimental effect.

The main limitation of life-course epidemiology of today is its failure to take a contextual approach into account, for example work-life [16]. The lack of contextualisation could be one reason why life-course epidemiology is much rarer in research on work life than e.g., in epidemiologic research on the impact of early individual risk factors. As a criticism of the fragmented research in the field, Amick et al. [16] conceptualised also work as life-course processes depending on place and time, with focus on labour market transitions and trajectories that various groups face during working life. They emphasize the need to understand the role of various contextual levels in shaping labour market and health trajectories to identify relevant policies and interventions. The relations between work and health are further complicated by the healthy worker effect, i.e., healthier employees are more likely to gain stable employments. Thus, health selection must be considered when analysing work-related exposures (e.g [8])..

To overcome the lack of context in life-course epidemiology we will contribute to theoretical development of the working life-course by integrating Elder’s [1] life-course theory with Urie Bronfenbrenner’s [3] Ecological Systems Theory of human development, which is of special importance from a life-course perspective since Bronfenbrenner’s theory can be used to understand how mental health develops from young age in close interplay with the larger social context. A central proposition in this theory is that people make choices and act in their social contacts as responses to emotional, cognitive, and behavioural elements of such interactions, and that these processes interact between settings on different ecological levels, conceived as a set of nested structures, each inside the next like a set of Russian dolls. Closest to the individual is the microlevel (the immediate settings including the family, the friends, the workplace, work conditions) followed by the mesolevel (the interconnections among several settings at the microlevel) and its extension called the exolevel, i.e., social structures which the person is not always actively involved in, or even present, but which are of major importance for their development, such as the neighbourhood. How society is organized on a “high” level is labelled the macrolevel (the overarching institutional patterns of gender relations, social class structure as well as the political, economic, legal, and social frameworks). The macrolevel permeates all the other levels. through different causal pathways, which is of key importance for the living conditions surrounding the individual.

Last, the chronolevel encompasses the temporal dimension and the gradual and often discrete changes of the context that occur over time, as well as the individual life history. This was the last level which Bronfenbrenner added to his model and has been least developed in research, even if time is implicit in all the processes described at the other levels of the model. Thus, the complexity of differential exposures across time and different life periods needs to be problematized. Incorporating an ecological approach into life-course epidemiology can provide a tool to analyse how determinants in various settings and ecological levels can interact and differ in extent, expression, and impact across the lifespan.

The aim of this study is to contribute to the theoretical development within the field of labour market effects on mental health by integrating Bronfenbrenner’s ecological model [3] with earlier theoretical work on life-course theory [1]. An integrative review will be performed, based on qualitative analyses of earlier published papers in the field of labour market and mental health in the Northern Swedish Cohort (NoSCo) in order to develop a contextualised life-course model. The research question is: How do labour market conditions explain the development of mental health over the life-course?

## Methodological and conceptual issues

### Material

The work of this paper has been performed within a large research programme called “Mental health in adolescence and the paths ahead. An ecological life-course approach to mental health development into adulthood”. We have performed an empirical analysis of all earlier publications about labour market conditions and mental health during life within the rich database of the Northern Swedish Cohort (NoSCo) [17]. The cohort had, at the time of the research programme, been followed over 27 years. For this qualitative metasynthesis, we chose all international publications in referee-judged international journals based on NoSCo about how mental health was related to labour market conditions (labour market attachment, work environment, school environment, students’ situation etc.). In total 52 papers were available and all of them were included in the analyse. Another integrative review of all papers about exposures outside paid work has been published from the same research programme [18].

NoSCo consists of all pupils in the last year of compulsory school in the municipality of Luleå during Spring term in 1981 (*n* = 1083). The cohort had at the time of the study been followed with questionnaires and health examinations at ages 16, 18, 21, 30 and 43. The participation rate has been extremely high – 94.3% of those alive at age 43 (*n* = 1071) participated. The cohort has been shown to be representative of the country in relation to socio-demographics and socioeconomic factors as well as in relation to health status and health behaviour. All young people who became unemployed directly after leaving compulsory school (*n* = 20) were included in an interview group and have been followed with personal interviews during the follow-up [17].

To analyse the health impact of the macrolevel, a so-called younger NoSCo was constructed [19]. The cohort population consists of all pupils in the last year of compulsory school in Luleå in 1989. They were first followed-up at age 21 (in the deep recession of 1994) and later at age 39 (in 2012). The response rate at age 39 was 85.6% of those still alive (*n* = 686). The comparison of the cohorts at age 21 indicate the impact of macroeconomic recession on young people’s health, as compared to the financial boom when the older group was 21 in 1986.

Two additional register-based populations were included in this paper. For each cohort participant, neighbourhood measures were collected in accordance with Statistics Sweden’s small-area market statistics (SAMS) [20]. SAMS is a small-scale geographic division constructed as polygons, using demographic distinctions (such as roads, buildings etc.) to demarcate neighbourhoods. In this study the SAMS areas were composed on 31 December in 1980, 1986, 1995 and 2007, consisting of at least one cohort member and on average 1000 individuals from the neighbourhood. The number of neighbourhoods increased from 72 in 1980 to 374 in 2007, due to the cohort members’ moving pattern.

Information was retrieved from Statistics Sweden for all residents living in each area. Unemployment was defined broadly as having the main source of annual income from unemployment benefits, health-related benefits and early retirement.

Register data for all cohort participants who worked at a workplace in Sweden at age 42 (*n* = 837) were collected. For each participant, register data about gender, salary, education, parental leave, temporary parental leave and age for all employees in each workplace (*n* = 135 398) was collected from Statistics Sweden [21].

#### Setting

Luleå is a medium-sized (with about 70,000 inhabitants) industrial town in Northern Sweden. The town is representative of medium-sized industrial towns in Sweden as regards the sociodemographic factors and labour market conditions except for doubled rate of youth unemployment during the early 1980ies as compared to the country as a whole. The labour market is dominated by manufacturing and mineral extraction, with a steel company and a large harbour. Other major empl:oyers are the public sector and a technical university. At the time of the start of the study in 1981, unemployment in the town was twice as high rate as in the rest of Sweden and there was more workforce immigration from Finland [22].

#### Dependent variable: mental health

There are inherent methodological challenges in the measurement of mental health problems in longitudinal research. There is constant development in definitions, taxonomies and demands concerning the properties of mental health measurements. We have tested the properties of the mental health measures (mostly single items) used when NoSCo was initiated in the early 1980ies in relation to the standards of today and conclude that composite measures of mental health problems can be constructed from single items which are more than 30 years old [23]. These measures seem to have the same factorial structure and internal consistency across a significant part of the life course.

Most papers in this metasynthesis used the composite measures of mental health described above consisting of depressive symptoms, anxiety symptoms and functional somatic symptoms (FSS). Depressive symptom score was based on six symptoms (sleeplessness, poor appetite, fatigue, concentration difficulties, feeling down or sad, and feeling downhearted about the future). The measure of functional somatic symptoms was constructed as a score of symptoms: headache or migraine, stomach ache (other than heartburn, gastritis or gastric ulcer), nausea, backache, hip pain or sciatica, general tiredness, breathlessness, dizziness, overstrain, sleeping problems, and palpitations. The measure of anxiety symptoms included five symptoms; restlessness, concentration difficulties, worries/anxiety, palpitations and spanic (for details of the variable construction, see Hammarström et al. [23]). Similar composite measures of mental health have been used in papers published before the validation paper. As externalised symptom, excessive alcohol consumption was used. A single measure of self-rated health is used in some papers and included as measures of depressiveness and anxiety as they have been shown to strongly contribute to self-rated health [24]. One paper used cortisol awakening response [58] as a measure of physiological stress.

#### Independent variable: labour market position

From 1983, matrices were developed in the questionnaires to measure various labour market positions (unemployment, labour market measures, temporary employment, studies etc.) each half-year period from the latest follow-up. Between age 16 and age 18, accumulated time in various labour market positions (studies, employment, unemployment respectively labour market measures),

Register data about yearly accumulated time in unemployment and labour market measures was available from 1992 and onwards.

#### Conceptual model and integrative review

The theoretical development during our research programme inspired us to develop a conceptual model which is here used for analysing and synthesising our material.

To obtain broader and more holistic knowledge regarding the mental health effects of working-life conditions over life, we have conducted a qualitative metasynthesis using the method of integrative review of papers about labour market conditions from NoSCo by adapting the Whittemore and Knafl’s framework [25].

Schick-Makaroff et al. [26]. have identified integrative reviews as one of several broad categories of research synthesis methodology. Integrative. reviews *“reviews, critiques, and synthesizes representative literature on a topic in an integrated way such that new frameworks and perspectives on the topic are generated”*, and *“ultimately present the “state of the art” of knowledge”* [26, page 198].

Integrative reviews summarize past empirical and theoretical studies and enables inclusion of studies with diverse methodologies. They can be used to address mature topics to re-conceptualize the expanding and diverse literature on the topic and to review new topics in need of preliminary conceptualization.

Whittemore and Knaufl [25] identified five steps in order to enhance rigour in integrative reviews: problem identification, literature search, data evaluation, data analyses and presentation. Problem identification was made in our large research programme with the aim of theory development and identification of how settings like labour market conditions explain the development of mental health over the life-course. Literature search was easily performed in relation to all international, referee judged publications about mental health and labour market conditions of NoSCo. The papers (including references) are presented in Tables 1 and 2. The final sample for this integrative review included both empirical (quantitative and qualitative) data and theoretical texts. The empirical data included were derived from a wide variety of methods: cross-sectional, various longitudinal quantitative methods, trajectory analyses, content analyses and theoretical development. Due to this diverse representation of primary sources, the publications were graded in the third step (data evaluation) according to methodological rigour as high or low. Five papers were coded as low rigour due to cross-sectional design [29, 31, 34, 50] and due to overadjustment [55]. No paper was excluded based on this evaluation, but these five papers contributed less to the analytic process.

#### Data analyses

The fourth step, data analyses of all included papers, was performed in the following way.

First, a deductive content analysis was performed of all papers in relation to Bronfenbrenner’s Ecological Systems Theory model [3]. Deductive implies that the analysed data was applied to a model and thus each paper was coded in relation to Bronfenbrenner’s ecological levels. Deductive approach represents a move from theory to data or from a more abstract and general to a more concrete and specific level. The main strength is that the theory that is used can be further developed and to some extent validated [27]. Each publication was coded with qualitative content analyses [28] in relation to the ecological levels of the model (see Tables 1 and 2). The statistical analyses in the various papers are controlled for baseline symptoms of mental health, i.e., for reversed causation, except in some of the early analyses (as stated in the paragraph above). These are stated to not control for baseline status in Table 1.

Each paper was coded into meaning units, i.e., condensed sentences in which the original content is preserved. Thereafter, the meaning units were sorted into descriptive categories, which answer the question ‘what’. Finally, categories with similar meanings were conceptualised into themes, which answer the question ‘how’.

Second, inductive qualitative content analyses [28] were performed. We coded how each paper captured the temporal dimensions, as any of the traditional life-course models or as other models. This coding resulted in several new time related concepts.

The meaning units, categories, and themes as well as the life-course pathways of each paper are shown in Table 1 (all quantitative papers) and in Table 2 (all qualitative papers). The results were ordered according to the Bronfenbrenner levels, beginning with the macrolevel and ending with the microlevel. The last two themes consist of results related to more than one level. In the fifth step (presentation), we present our findings in Tables 1 and 2 and developed a contextualized life-course model based on mainly the themes from these tables (see figure below).

In developing our model, we used the levels suggested by Bronfenbrenner [3] and allocated them with our empirical findings (see Tables 1 and 2). We filled the time dimension (the chronolevel) with time related processes from both predefined life-course models [15] and with processes derived from the inductive part of our analysis. Our main contribution was combining Bronfenbrenner’s model and life-course epidemiology and thereby adding empirical data to the settings on various levels and new processes to the temporal dimension.

The trustworthiness of our findings was achieved according to scientific tradition in qualitative research [27]. Credibility of our research findings deals with how well themes and meaning units cover data, In the analyses of the included papers, no relevant data have been systematically excluded or irrelevant data included. To increase the trustworthiness of the study, the interpretations and formulations of themes were discussed and negotiated among the authors during the process of analyses. All meaning units were covered in each theme, which increases the trustworthiness. In order to ensure transferability, the setting of the study has been described in detail in both the Method and the Results and Reflection settings.

## Results and reflections

The findings are presented in detail in Tables in relation to each Bronfenbrenner level as well as in relation to predominant life-course pathways. Below we concentrate on making a more holistic view on the results. Some themes permeate several levels while others are mainly situated on the microlevel.

First, the Bronfenbrenner model is described in more detail [3]. The *macrolevel* can be defined as how society is organized on a high level including structural relations (in relation to class, gender, ethnicity etc.), laws, institutions, policymaking as well as functional and normative elements (e.g., religiose norms, prejudices) [3]. Is the result of various policy making processes). Structures and processes on the macrolevel permeate all the other levels of the ecosocial system and are finally embodied through the effect of specific individual experiences resulting from those structures and processes, ultimately leading to different health outcomes. These embodied experiences are incorporated into the body from past time into the present in terms of susceptibility or resilience in relation to “exposures” on various level.

The *exolevel e*mbraces major arenas of society—such as the world of working life and the neighbourhood. These social arenas do not themselves contain the developing person but affect the immediate settings in which that person is found, and as such influence what is going on in these settings [3]. The *mesolevel* constitutes the interconnections among the settings at the microlevel, while the microlevel includes the settings where the individual directly interacts with others, such as the family, the friends, the workplace. The *microlevel* consists of the contexts where an individual interacts directly with other individuals, e.g., family, workplace, network of friends, etc.

Second, the results on and reflections about all nine themes of the qualitative analyses (Tables 1 and 2) are presented. The last two themes consist of results related to more than one ecological level.

**Table 1:** Analyses of quantitative papers related to work and mental health in the Northern Swedish Cohort. (Labour market attachment = LMA)

Levels	Meaning units	Categories	Theme	Predominant life-course pathways
Macro	Those 21-years old who studied during recession had more depressive and functional somatic symptoms than those who studied during boom. No long-term effect of recession was observed at the age of 39/43 [30].	To be exposed to recession while studying is related to poor mental health. No long-term effects.	1.Macroeconomic recession impairs mental health among young people.	Sensitive period
Macro	Cross-sectional analyses of not long-term unemployed young people found poorer mental health in recession compared to boom among students and for women also among those in work and in labour market measures [31]. No long-term effects were studied. Baseline mental health could not be controlled for (in younger NoSCo).	Mental health is worse among 21-years old (particularly among women) who were not long-term unemployed during recession compared to boom.	1.Macroeconomic recession impairs mental health among young people.	Cross-sectional analyses
Macro	Alcohol use did not differ by trade among 21-year-old [32].	Macroeconomic conditions do not affect the use of alcohol among young people.	2. The mental health effects of youth unemployment seem rather insensitive to recession.	Sensitive period
Macro	Macro level recession did not modify the relation between accumulation of time in unemployment from the age 21 to 25 and mental health symptoms in adulthood. No short-term effects were analysed [33].	The health effects of exposure to youth unemployment on adult mental health seem to be rather insensitive to recession.	2. The mental health effects of youth unemployment seem rather insensitive to recession.	Sensitive period and accumulation
Macro	21-year-old long-term unemployed had the same level of somatic and psychological symptoms during boom compared to recession [34]. Baseline mental health could not be controlled for.	The health status of long-term unemployed youth does not differ between boom and recession	2. The mental health effects of youth unemployment seem rather insensitive to recession.	Cross-sectional analyses
Exo	Living in a neighbourhood with high unemployment has implications for residents’ level of functional somatic symptoms, regardless of their own unemployment across time, particularly at age 30 [35].	Neighbourhood unemployment is negative for individuals’ health, independent of their own employment status.	3. Small but consistent negative effect of neighbourhood unemployment and other work-related disadvantages over life on mental health	Accumulation, sensitive periods
Exo	Cumulative neighbourhood disadvantage between ages 16 and 43 years was related to higher allostatic load at age 43 years (after adjusting for personal living conditions) among men [36].	Cumulative neighbourhood disadvantage during life is related to biological wear and tear among men.	3. Small but consistent negative effects of neighbour-hood unemploy-ment and other work-related disadvantages over life on mental health.	Accumulation
Exo	Neighbourhood disadvantage seems to have greater importance for mental health in young age, and thereafter diminish. The impact of individual material and social resources (including unemployment) increase with age [37].	Neighbourhood seems to have greatest importance for mental health in young age. Material and social adversity became increasingly important as one grew older.	3. Small but consistent negative effect of neighbour-hood unemploy-ment and other work-related disadvantages over life on mental health.	Sensitive period Accumulation
Exo	Accumulation of both individual and neighbourhood disadvantages from youth until midlife was related to adult mental ill health [38].	Cumulative effect of neighbourhood disadvantages on adult mental health.	3. Small but consistent negative effect of neighbourhood unemployment and other work-related disadvantages over life on mental health.	Accumulation
Exo	Living in a disadvantaged neighbourhood context in adolescence was among boys related to alcohol consumption later in life. This is not completely explained by adverse family circumstances in adolescence [39].	Neighbourhood disadvantage in adolescence increases the risk for later alcohol consumption.	3. Small but consistent negative effect of neighbourhood unemployment and other work-related disadvantages over life on mental health.	Sensitive period
Exo	Results from cross-classified multilevel models showed that 12.7% of age 42 mental health variance was explained by an interaction of age 16 and age 42 neighbourhood of residence [40].	Mental health variation by neighbourhood in mid-adulthood may partly depend on neighbourhood of residence in adolescence.	3. Small but consistent negative effect of neighbourhood unemployment and other work-related disadvantages over life on mental health.	Sensitive periods
Exo	Socio-economic and psychosocial factors in midlife explain a substantial part of neighbourhood inequalities in mental health. Also, the inequality can originate from conditions in adolescence and young adulthood [41].	Socio-economic and psychosocial factors in midlife are important to understand neighbourhood inequalities in adult mental health.	3. Small but consistent negative effect of neighbourhood unemployment and other work-related disadvantages over life on mental health.	Sensitive periods
Exo	Adolescent neighbourhood disadvantage, family, and school circumstances but not health nor health behaviour were independently predictive of the socioeconomic character of one’s neighbourhood of residence in mid-adulthood [41].	No health selection into neighbourhood disadvantage.	3. Small but consistent negative effect of neighbourhood unemployment and other work-related disadvantages over life on mental health.	Sensitive period
Macro Micro	In both boom and recession, unemployment from age 21 to 25 was related to mental health symptoms (anxiousness and depressiveness) in middle age. No differences were found in relation to trade [33].	The health effects of youth unemployment on adult mental health seem to be rather insensitive to the recession.	2 and 4. Youth unemployment becomes embodied as scars of mental ill-health over life.	Sensitive period and accumulation
Micro	Accumulated time in youth unemployment (age 18–21) was related to mental health symptoms at the ages of 21, 30 and 43. Those exposed to three periods of unemployment had the worst mental health development [42].	Scarring effects of youth unemployment.	4. Youth unemployment becomes embodied as scars of mental ill-health over life.	Sensitive period and accumulation
Micro	Among men, accumulated time in youth unemployment (age 16–21) was related to functional somatic symptoms at age 21 The relation remained at age 42 regardless of later unemployment (age 21–42) [43].	Scarring of youth unemployment on functional somatic symptoms among men	4. Youth unemployment becomes embodied as scars of mental ill-health over life.	Sensitive period and accumulation
Micro	Accumulated time in youth unemployment (age 16–21) among young men and women was related to smoking, nervous and depressive symptoms and – among men only – somatic symptoms at the age of 30 regardless of parental class, early health, and later unemployment (age 22–30). No relations for alcohol consumption [44].	Scarring of youth unemployment on mental health but not on alcohol consumption later in life.	4. Youth unemployment becomes embodied as scars of mental ill-health over life.	Sensitive period and accumulation
Micro	Unemployment in early adulthood is related to later -ill-health independent of later unemployment [45].	Scarring of unemployment in early adulthood on poor self-rated health	4. Youth unemployment becomes embodied as scars of mental ill-health over life.	Accumulation Sensitive period
Micro	Accumulated time in youth unemployment (age 16–21) particularly among men was related to high levels of alcohol consumption throughout adulthood [46].	Youth unemployment is related to high levels of alcohol consumption over life.	4. Youth unemployment becomes embodied as scars of mental ill-health over life.	Sensitive period and accumulation
Micro	Accumulated unemployment but not accumulated participation in youth programs between age 18 and 21 was related to adult mental health [47].	Scarring of unemployment in early adulthood on adult mental health. Youth programmes seem to be protective of scarring.	4. Youth unemployment becomes embodied as scars of mental ill-health over life.	Sensitive period and accumulation
Micro	More somatic and psychological symptoms and higher alcohol consumption was found at age 21 among long-term unemployed compared to other groups [48].	Youth unemployment is related to poor physical and mental health and health behavior in younger age.	5. Weak LMA impairs mental health over life.	Accumulation
Micro	Accumulated time in youth unemployment (age 16–18) was related to psychological symptoms and increased use of alcohol. A theoretical model showed the importance of lack of control in compulsory school on both unemployment and mental health outcomes [29]. Baseline alcohol consumption (but not mental health) was controlled for.	Youth unemployment is related to mental health and high alcohol consumption in young age.	5. Weak LMA impairs mental health over life.	Accumulation
Micro	Dose-response correlations were found between accumulated time in unemployment (age 16–30) and 1.health behaviour among men 2. Symptoms of mental health among women [49].	The longer the exposure to unemployment from youth to early adulthood, the worse the mental health (among women) and the health behaviors (among men).	5. Weak LMA impairs mental health over life.	Accumulation
Micro	The association between accumulated time in unemployment (during the last 5 years) and mental ill-health was stronger in young people than in adults [50]. Baseline health was not controlled for.	Young people seem to be harder hit by unemployment than older.	5. Weak LMA impairs mental health over life.	Sensitive period and accumulation
Micro	Accumulated time in unemployment between 40–42 was related to mental health as well as health behavior (alcohol consumption among women and fewer dentist visits among men) [51].	The negative health effects of unemployment are more similar than different between men and women.	5. Weak LMA impairs mental health over life.	Accumulation
Micro	Dose response relations between youth unemployment and increased nervous and depressive symptoms (similar among men and women). Qualitative methods identified mediating factors such as lack of self-confidence, self-blame, stress, isolation, lack of control and resignation [52]	Accumulated time in unemployment is related to impaired mental health. Possible mediating mechanisms.	5. Weak LMA impairs mental health over life.	Accumulation
Micro	Accumulated time in youth unemployment (age 16–21) was related to higher alcohol consumption (both average consumption and percentages with high consumption) among young men (age 21) [53]	Irrespective of earlier alcohol consumption, youth unemployment leads to increased alcohol consumption among young men.	5. Weak LMA impairs mental health over life.	Accumulation
Micro	Temporary employment at the age of 42 was associated with self-rated health and psychological health. Low cash margin and job insecurity may partially mediate the association [54].	Temporary employment is related to poor mental health in adult life.	5. Weak LMA impairs mental health over life.	Lifetime analysis controlled for earlier mental health
Micro	Highest exposure of accumulated time in the most peripheral employment position over a 12-year period (age 30–42) was related to higher odds of psychological distress in midlife (age 42) ) [55]. Among men the results were significant in all models. No model controlled only for earlier health. Therefore, the lack of significant results among women (except in the unadjusted model) needs to be interpreted with caution due to overadjustment.	Exposure to the most insecure and short-term employment contracts in adulthood are related to impaired mental health.	5. Weak LMA impairs mental health over life.	Accumulation
Micro	Accumulated time in nonpermanent employment from age 30 to 42 was associated with nervous symptoms, psychological distress, and suboptimal mood at age 42. No effects were found in relation to somatic health and health behaviors [56].	Temporary employment in adulthood is related to poor mental health but not to alcohol.	5. Weak LMA impairs mental health over life.	Accumulation
Micro	Weak trajectories of LMA from age 30 to age 43 were related to psychological distress, independently of social background and previous health [57].	Weak LMA is related to mental ill-health	5. Weak LMA impairs mental health over life.	Accumulation
Micro	Accumulated time in temporary employment from the age of 30 to 42 was associated with hypothalamic-pituitary-adrenal axis dysregulations in the form of increased dynamics of the cortisol awakening response and circadian suppression at the age of 43 [58].	Temporary employment is related to disturbances of physiological stress system.	5. Weak LMA impairs mental health over life.	Accumulation
Micro	Accumulated time in temporary employment (age 30 to 42) was related to lower self-related health at age 42 in participants with lower education [59].	Low educated are most badly hit by the mental health consequences of temporary employment.	5. Weak LMA impairs mental health over life.	Accumulation
Micro	Obtaining permanent employment after an unstable labor market position at the age of 25–30 was related to lower probability of having psychological symptoms at the age of 30 [60].	Transition from an unstable labor market position to permanent employment is related to better mental health.	5. Weak LMA impairs mental health over life.	Lifetime analysis controlled for earlier mental health
Micro	Poor work characteristics (higher demands, lower job control, and lower support) were related to poor self-reported health and emotional exhaustion at the age of 43 [61]. Low job control was seen among short-term employed.	Short-term job contracts are related to low job control which in turn impairs mental health.	5. Weak LMA impairs mental health over life.	Lifetime analysis controlled for earlier health
Micro	Exposure to accumulated job insecurity between (age 30–42) was related to suboptimal mental health at the age of 42. The effect of job insecurity on health was the same in both permanent and temporary employees [62].	Accumulation of job insecurity is related to poor mental health in people with both temporary and permanent employment.	5. Weak LMA impairs mental health over life.	Accumulation
Micro	Lack of professional establishment (being retired, unemployed, in labour market measures) in early adulthood can mediate the association between adolescent school connectedness and depressive symptoms in adulthood [63].	The relation between poor school situation and adult depressiveness could partly be explained by lack of professional establishment in early adulthood.	5. Weak LMA impairs mental health over life.	Sensitive period
Micro	Getting out of locked-in situation was related to improved and getting into locked-in with worsening mental health between age 30 and age 43. The worsening was more pronounced and the improvement less pronounced in white-collar than in blue-collar employees. Poor mental health at age 16 predicted locked-in situation in early middle age [64]	Increasing looked-in is related to impaired while decreasing was related to improved health.	5. Weak LMA impairs mental health over life.	Social mobility
Micro	Poor mental health at age 16 predicted locked-in situation in early middle age [64]	Poor mental health at age 16 is related to becoming locked-in.	6. Bidirectional relations between health and weak LMA over life	Social mobility
Micro	Longstanding disease, symptoms (except psychological symptoms among girls), smoking and alcohol consumption at age 16 did not influence the risk of unemployment five years later [65].	Almost no early health selection into youth unemployment.	6. Bidirectional relations between health and weak LMA over life	Lifetime analyses
Micro	Depressive symptoms in youth were not related to life-course trajectories (measured as sequence analyses) of education and LMA among men and women. Unemployed parents were strongly related to weak attachment [66]	No health selection into weak LMA	6. Bidirectional relations between health and weak LMA over life	Accumulation
Micro	Suboptimal health status and health behavior at the age of 30 predicted both the occurrence of unemployment and, in particular, prolonged unemployment in early middle age (31–42) irrespective of unemployment earlier in the life-course [67].	There is health-related selection into unemployment in early middle age, irrespective of unemployment earlier in the life-course.	6. Bidirectional relations between health and weak LMA over life	Lifetime analysis adjusted for earlier mental health
Micro	Suboptimal self-related health and smoking at the age of 30 was related to high accumulation of non-permanent employment from the age of 30 to 42. More health selection was found among women [68].	Self-related health predicts stability of employment, and the selection is stronger among women.	6. Bidirectional relations between health and weak LMA over life	Lifetime analysis adjusted for earlier mental health
Micro	Heavy episodic drinking at ages 16 and 30 in men (and at age 21 in women) were related to subsequent unemployment up to midlife [69].	Heavy episodic drinking is related to later unemployment	6. Bidirectional relations between health and weak LMA over life	Lifetime analysis controlled for earlier mental health
Micro	Depressive symptoms in adolescence influence later unemployment in adult age. Lack of social support at age 30 is a mediator [70].	There is health-related selection into unemployment, mediated via poor social support.	6. Bidirectional relations between health and weak LMA over life	Accumulation
Macro Micro	Determinants of intergenerational mobility (between parental - and own social class) and intragenerational mobility (between own social classes during life) were analysed. Being less popular at school predicted downward social mobility. Additionally, material deprivation, economic deprivation, shorter height (women) and poor health behaviour predicted downward mobility [72].	Apart from height (women), health status is not associated with social class mobility either inter- or intergenerationally. Unfavourable school environment was a consistent determinant of negative mobility for both genders.	7. Macrolevel structures of importance for how labour market position caused poor health.	Social mobility
Macro Micro	Earlier mental health had no impact on working-class related musculoskeletal disease at age 30 while poor work environment (job control and most of all physical heavy job) had the strongest impact on women. Among men earlier alcohol consumption was also important [73].	No mental health selection into class-related musculoskeletal disease.	7. Macrolevel structures of importance for how labour market position caused poor health.	Sensitive period Accumulation
Macro Exo Micro	Parental working-class status was related to poor mental health in adulthood, partially mediated through unfavourable material and social conditions during life, ultimately affecting health in adulthood [74]	Growing up in an unfavourable socioeconomic circumstance can initiate a chain of risk of adverse living condition, which in turn affects adult health.	7 Macrolevel structures of importance for how labour market position caused poor health.	Chain of risk
Macro Micro	The socioeconomic gradient’s effect on health at age 30 could be explained by labor market experiences including accumulated time in unemployment (age 16–30) and physically heavy work [75].	Accumulated unemployment as well as physical heavy work could explain the effect of the socio-economic gradient on mental health in young adulthood.	7 Macrolevel structures of importance for how labour market position caused poor health	Accumulation
Macro Micro	Job strain was related to increased allostatic load at the age of 43 only among participants with adversity in adolescence [76].	Early adversities modify the relation between job characteristics and biological stress.	7. Macrolevel structures of importance for how labour market position caused poor health.	Sensitive period and susceptibility
Macro Micro	Despite similar disadvantaged background, the participants in youth opportunity programs had the largest decrease in mental health symptoms and alcohol consumption as compared to the unemployed group [29]	Participation in youth opportunity programs is beneficial for health among teenagers.	4,7. Macrolevel structures of importance for how labour market position caused poor health.	Accumulation
Macro Micro	Strong and significant relation between accumulated time in youth unemployment (age 18–21) and internalized mental health symptoms was observed at age 21 and 43. No such significant relation was observed for exposure to youth programs at age 21 or 43 [47].	Active labour market policies directed towards youths could potentially reduce the short- and long-term mental health costs of youth unemployment.	4,5 and 7. Macrolevel structures of importance for how labour market position caused poor health.	Sensitive period and accumulation
Macro Exo	Traditionally gender unequal workplace was related to psychological distress among women at the age of 43. The lowest overall occurrence of psychological distress as well as same occurrence for women and men was found on the most gender equal workplaces [21].	Workplace gender equality is related to mental health among men and women.	8. Unequal gender relations at various levels impacts negatively on mental health.	Lifetime analysis adjusted for previous mental health

### Macroeconomic recession impairs young people’s mental health

During the 1980s the unemployment rates were high in Northern Sweden as compared to the rest of the country [22]. Local policies were developed to tackle the problem, by incentivising the unemployed in this region to move south [29]. The sudden recession in the early 1990s hit the whole country hard, with sharply increased levels of unemployment in the country at large [19]. Men-dominated workplaces, such as manufacturing industries, were affected first, followed in the mid-decade by cutdowns in the women-dominated public sector [19]. After the recession of the 1990s, the levels of unemployment decreased in the country, but never to earlier levels. The so-called great recession 2007–2009, by comparison, was relatively mild in Sweden.

Comparing mental health among 21 years old in relation to labour market position (excluding long-term unemployed) in boom and recession we found more symptoms among students during recession but otherwise no differences among young men [30]. Young women in work and in labour market measures had worse mental health during recession than during boom [30]. An explanation could be that in women-dominated, as compared to men-dominated, sectors, the workload cannot necessarily be reduced when cut-downs in the staff are made [31]. Thus, the work environment becomes more strained, when fewer staff are expected to produce the same or even larger volume of work in health care, social care, and education.

The business cycle-related differences in mental health among 21-year-old students did not last until adult age [30]. During life the mental health of those who were students during recession may have improved thanks to their ’forced’ studies: the education could have started a positive chain of possibilities, leading to more qualified jobs with better salaries and improved work environment, compared to those who entered the labour market [30].

### The health effects of youth unemployment on individuals do not differ due to the trade cycle

The long-term consequences of youth unemployment on adult alcohol consumption [32]. and mental health [33] (scarring) were insensitive to the trade cycle. In other words, it seemed to be just as bad for mental health among 21-years old to be unemployed in boom as in recession [34].

### Small but consistent negative effect of neighbourhood unemployment and other work-related disadvantages over life on individuals’ mental health

Several publications dealt with the negative impact of neighbourhood disadvantage on inhabitants’ mental health [20, 32, 35–41]. During the 1990s recession, the exo-level phenomena of high rates of unemployment in the neighbourhood were related to impaired mental health, independently of the individual’s own labour market status [35]. High area unemployment could indicate high degree of marginalization among inhabitants and an environment, characterized by deprivation in infrastructure, educational and labour market opportunities, availability of healthy foods at affordable prices, increased stress, and lack of social support. Living in deprived areas could therefore increase individual strain, independent of a person’s own position in the labour market [35]. In addition, cumulative neighbourhood disadvantage (defined as not working, low income, lack of wealth, housing allowance and low socioeconomic status) during life was related to biological wear and tear among men [36] and poor mental health among both genders [37]. Neighbourhood disadvantage seems to have its greatest importance for mental health in young age [38]. Neighbourhood disadvantage in adolescence increases the risk for later alcohol consumption [39].

One of our studies analysed possible predictors of adult neighbourhood disadvantage. Adolescent neighbourhood disadvantage, family, and school circumstances but not health nor health behaviour were independently predictive of the socioeconomic character of one’s neighbourhood of residence in mid-adulthood. Thus, no health selection into neighbourhood disadvantage was found [20].

### Youth unemployment becomes embodied as scars of mental ill-health over life

Several publications analyse the scarring effect of youth unemployment on mental health over life. In this context, scarring means that, while youth unemployment has well-known direct effects on health (“wounds”), these wounds remain as scars in adult age (measured in relation to alcohol consumption and various measures of mental health) [33]. Scarring thus denotes adversities due to individual exposure to unemployment that remain - or become - ‘visible’ when the individual has passed the actual unemployment episode. In other words, the health effects that remain after the exposure to unemployment has ended [33].

While mental health and alcohol scarring was consistently shown for youth unemployment [33, 42–46], no such scarring was found from participating in Youth opportunities programmes [47].

### Weak labour market attachment over life impacts on mental health

The direct negative health effects of exposure to accumulated unemployment, especially in young, but also in adult age, have been widely documented in our data in relation to symptoms of mental health [17, 19, 22, 29, 34, 48–52] as well as to excessive alcohol consumption [53]. Cumulative exposure to unemployment (as little as 12 weeks between ages 16 and 18 years [29], and 6 months between ages 16 and 21 years [44] is related to impaired health and health behaviours in young age. The longer time of exposure to unemployment, the worse the health outcome [49, 52]. Men and women are equally hit by the health consequences of unemployment, although the health expressions may differ [51].

The research also shows that having temporary employment contracts in young adulthood impacts negatively on mental health in adulthood [51, 54–62]. The findings could be mediated via poor financial situation and job insecurity [54]. Moreover, the health effects of non-permanent employment depend on the socio-economic status of the employees, being more adverse in those with short education [59].

Transition from an unstable labor market position to permanent employment is related to improved mental health [60]. Being unemployed or out of the labour market at age 30 could explain the relation between poor school situation and adult depressiveness [63].

Locked-in on the labour market (having permanent contract in a non-preferable workplace or job) may be regarded as a kind of weak position on the labour market and is related to deteriorated mental health [64]. Getting locked-in increased mental symptoms over a ten-year period, while getting out of such situation improved mental health.

### Bidirectional relations between health/behaviour and weak labour market attachment over life

The possible bidirectional relations between health/ health behaviour and later labour market attachment were analysed in almost all papers, by controlling for health/ health behaviour before exposure to the labour market. As it is well known that poor health and unfavourable health behaviours increase the risk of weak position on the labour market (so-called health-related selection), Several of our papers focused on the exposure effects, controlling for health selections [62, 65–70].

An interesting finding is the almost lack of or weak effect of health selection -especially in young age – into later unemployment [52, 65]. In fact, some of our research shows that the effects of exposure to unemployment in young age are stronger than the health-related selection [53].

Even with follow-ups until age 43, depressive symptoms in youth were not related to life-course trajectories (measured as sequence analyses) of weak labour market attachment [66].

Later in life, in young adulthood there is health-related selection into both unemployment [67] and temporary employment contracts [68], irrespective of exposure earlier in life. The findings may be interpreted as an indication of discrimination against those with mental health problems in the labour market.

### The combination of macro- and other levels are of importance for how labour market position cause poor health

The theme includes macrolevel structures of major importance for how the labour market position can cause poor mental health over life. These structures include the construction of social class, which stratifies individuals into hierarchal social positions in relation to education, income, and occupation. These structures are powerful determinants of individuals life chances and health status during life [71].

Unfavourable school environment (rather than health) was a consistent determinant of negative inter- and intragenerational mobility between social classes [72]. Growing up in an unfavourable socioeconomic circumstance can cause a chain of risk of adverse living condition, which in turn affects adult mental health [73].

Accumulated unemployment as well as physical heavy work could explain the socio-economic gradient in self-rated health in young adulthood [75]. Early social adversities modify the relation between job characteristics and biological stress [76]. Thus, early disadvantages can have a long-term impact on vulnerability to work stress.

Macrolevel policies for facilitating the transition of unemployed into the labour market programmes have been in focus in our research. We conclude that there is a lack of research in the field, but a considered and consistent active labour market policy directed at youths could potentially reduce the short- and long-term mental health costs of youth unemployment [29, 47].

Being in youth programmes between age 16 and 18 seem to be health promoting, compared to being unemployed. During the two-year follow-up, especially girls (and in relation to health behaviour also boys) in youth opportunity programmes improved their mental and health behaviour while mental health was impaired among boys and girls in unemployment [29]. Thus, youth opportunity programmes during this period of life seems to be health promotive.

### Unequal gender relations at various levels impacts negatively on mental health

The societal gender order, constructed by multiple ideas about what is seen as feminine or masculine in a certain historical and societal context, is useful for understanding the gendered working conditions and its importance for health over life [77]. The gender order is built up by unequal gender relations, which permeates from the macro- to all other levels and creates the gender-segregated labour market, which divides men and women into different sectors and occupations as well as into gendered hierarchies.

The importance of gender equality at workplaces has been analysed on the exolevel in adulthood [21]. All employees at the workplaces, in which one or more participants worked, were included in the analyses. Register data was available, so a measure of gender equality was constructed (based on the proportion of men respectively women and the gender gradient in education, income, sick-leave, and parental leave), and analysed in relation to each workplace in which at least one participant in NoSCo worked. A cluster analysis resulted in six distinctive clusters with different patterns of gender equality at the workplaces that were associated to psychological distress among women [21]. For women the highest odds of psychological distress were found on traditionally gender unequal workplaces. The lowest psychological distress was found on the most gender equal workplaces, for both men and women. Thus, workplace gender equality was related to good mental health among both men and women.

### The agency to improve health over life in dyadic relations in various microlevel settings

Agency refers to how individuals act within (or despite) social settings [78]. Thus, individuals can use agency to negotiate, challenge and transform their contexts and the surrounding intersecting power-structures, related to for example gender and social class.

The qualitative papers (see Table 2) illustrate how the participants constructed gender and developed agency in relation to their health over life. 

**Table 2:** Analyses of qualitative papers related to health, work, and labour market in the Northern Swedish Cohort

Level	Meaning unit	Categories	Theme	Predominant life-course pathway
Individual, Micro, Macro	The hardworking masculinities strived to fulfil the masculine societal ideal of working-class men in society by accepting all available jobs, despite hazardous work environments in jobs that were available for uneducated men on the labour market. Workplace accidents were common. In contrast, the disconnected masculinity was related to disposition of pessimism and meaninglessness, as part of the young men’s scarce beliefs in having a future due to their marginalized position on the labour market. This masculine position could also be expressed in criminality or drugs use. A more health-promoting masculine position was identified as connected masculinity, of caring and protection through hard work, in line with the collectively oriented welfare state ideas about responsibility for those unable to take care of themselves [79].	A model of agency within structures was useful for and to capturing the agency and intentions of participants in the analyses of disposition to act in relation to health	8. Unequal gender relations at various levels impacts negatively on mental health. 9. The agency to improve health over life in dyadic relations.	Developing agency over life
Individual Micro Meso Macro	A follow-up of all the girls in disfavored social position (who became unemployed directly after leaving school) until age 33 showed that the more they kept to the normative and altruistic femininity, the more social support and health they gained, while challenging this ‘essentialized’ or ‘patriarchal’ way of doing femininity or failing to conform was related to lower social support and more health problems [80].	Femininities and health related experiences were constructed in relation to marginalization and social context	8. Unequal gender relations at various levels impacts negatively on mental health 9. The agency to improve health over life in dyadic relations	Developing agency during life
MacroMicro	Health experiences can be viewed as a contextual process, related to the different phases of leaving school, entering the labour market, becoming unemployed and becoming employed. Perceived relief and hope were related to leaving compulsory school, while entering the labour market was related to setbacks and disappointments as well as both health-deteriorating and health-promoting experiences depending on the actual labour market position. Our overarching theme of “Living in the shadow of unemployment – an unhealthy life situation” implies that not only the actual situation of being unemployed is problematic but the other phases are also colored by macrolevel unemployment [81].	Macrolevel unemployment influences the health of both unemployed and employed.	1, 4 and 9. The agency to improve health over life in dyadic relations in various microlevel setting	Developing agency during life
Individual, Micro, Meso, Macro	Among women, we identified gender relations in relation to ‘constructing respectability from disfavoured social positions’. Within this position, framed by dominant norms of patriarchal femininity, we explored the constructs of the agentic and health promoting normative and altruistic femininity as compared to the norm-breaking and risk-taking femininity related to impaired health and health behaviour [80].	Constructing respectability from disfavoured social positions	8. Unequal gender relations at various levels impacts negatively on mental health. 9. The agency to improve health over life in dyadic relations.	Developing agency over life
Individual, Micro Macro	One unemployed young man, who had become depressed due to a most problematic family situation, felt ‘perfect’ when he could enter a youth programme and work at a garage mending cars. Other young men described themselves as too lazy to contact the Employment Office. “…It was meaningless to go to the employment office: First of all, I am too lazy. It takes such a long time before I get the unemployment benefit. You must work half a year to the benefits. Then I believe that it is meaningless. Total meaningless.. I have not been there [Employment Office].and will not put my foot there.” One young man expressed his healthier habits when moving in with his girlfriend in the following way; ‘And when you got a girlfriend then you have to calm down a little’. [79].	Dyadic relations between the individual and microlevel settings.	8. Unequal gender relations at various levels impacts negatively on mental health. 9. The agency to improve health over life in dyadic relations.	Developing agency over life

A model of agency within structures was useful for analysing disposition to act and to capture the will and intentions of early unemployed men in relation to health [79]. The hardworking masculinity type of men developed agency by striving to fulfil the masculine ideal of working-class men in society by accepting all available jobs, despite hazardous work environments in jobs that were available for uneducated men on the labour market. Workplace accidents were common. In contrast, less agency was found in relation to the disconnected masculinity, which was related to development of pessimism and meaninglessness, as part of the young men’s lack of belief in having a future due to their marginalized position on the labour market. This masculine position could also be expressed by criminality or drug abuse. A more agentic and health-promoting masculine position was identified as connected masculinity, of caring and protection through hard work, in line with the collectively oriented welfare state ideas about responsibility for those unable to take care of themselves [79].

Among early unemployed women, we identified gender relations as ‘constructing respectability from disfavoured social positions’ [80]. Within this position, framed by dominant norms of patriarchal femininity, we explored the constructs of the agentic and health promoting normative and altruistic femininity as compared to the norm-breaking and risk-taking femininity related to health and health behaviours that became deteriorated.

Dyadic and complex relations were identified between unemployed persons and others in various settings at the microlevels, such as employment officers, employers, parents, and partners [81]. The macrolevel policies of moving to the South of Sweden influenced the relations between the unemployed youth and the employment officers. The young unemployed and their parents were upset about the advice for a 16-year-old to move alone far away and alone to a large city.

Relations with employers could be negative in terms of rejected job applications, getting insecure job contracts or no prolongation of contract [81]. The relations could also be very positive. Getting a job was felt as becoming a valuable citizen, given the possibilities of doing something valuable. A job could mean belonging to a team at the workplace, to be treated with respect and expected to be a capable worker. Having a job also lead to restricting late nights out drinking.

The macrolevel gender order shape relations in family so that the girls, but not the boys, were expected to do domestic duties while unemployed. Gendered caring responsibilities meant that becoming a mother was experienced as being a respectable position in society [80]. In addition, unemployed young men described how a girlfriend could be an important support for calming down after having lived a risk-taking life in relation to alcohol [79]. A girlfriend was described as important because you could talk to her; she gave hope to your life and could make you feel less restless. No similar stories about the positive impact of a boyfriend were told by the unemployed young women [79].

For young unemployed, relations to parents could be ambivalent [81]. They could be economic dependent on parents. But parents could also helpful. The young unemployed men could experience support from parents, e.g., help to find a job, financial support or to live with parents to avoid living a dangerous life.

A qualitative paper identified what macrolevel unemployment can mean for young people in work. “Living in the shadow of unemployment – an unhealthy life situation” implies that all young people may be influenced by the high rates of unemployment in society [81]. You may be forced to study as there are no jobs available. Also, the situation as employed is also coloured by the rate of unemployment in society. Due to lack of jobs and job insecurity you must take any job available despite poor work conditions and lack of safety regulations.

The paper also explored which possible mechanisms could explain how high levels of societal labour market processes could permeate down to the microlevel and cause scarring in the individual [81]. Negative feelings related to unemployment in young age – such as disappointment, dependence, distrust, and resignation – could become embedded in the mind and in the patterns of reactions to stressful life-events later in life rather than stir an active engagement against injustice and disappointments in life. Such negative feelings are closely embedded in depressive states and may be a key to our understanding of unemployment scarring.

## Temporal dimensions

The life-course was mainly analysed according to the model of accumulation of exposure and, in relation to scarring also as models of sensitive periods [15]. Scarring per se can also be viewed as a temporal dimension, viewing the past being present as a permanent functional structure in the individual. Our use of trajectory analyses are also temporal methodological dimensions from past to present.

We developed the concept “lifetime analyses, controlled for earlier mental health” based on studies with mainly cross-sectional analyses, controlling for earlier health.

In the qualitative analyses, we identified a novel temporal dimension in relation to agency. The concept of agency is strongly linked to time because it represents a process that evolves over time. For example, if you lose your job, it takes time to respond to this in an agentic manner. It takes even longer time for the effect of the executed agency to manifest itself on the mental health of the individual for example when being employed again. The possible latitude of an individual’s agency is determined by several factors in the ecological system that may restrict it, such as macrolevel factors like inequality due to gender, social class and ethnicity that permeate all levels of the system. Factors which could facilitate agency include legislation or interventions in different social arenas such as labour market policies with the purpose of countering the mentioned agency-restricting phenomena. These circumstances further support the need of a complex integrative theoretical framework based on a system approach and with a strong notion of time as a fundamental element.

## Concluding remarks

Mental health over life is shaped by the macrolevel structures of society defined by Bronfenbrenner and the labour market context surrounding the individual on both close and more distant levels. Neighbourhood disadvantages have a small but consistent negative impact on the individual’s health, especially in young age. Weak labour market attachment negatively impacts the mental health of the individual over life, in addition to bidirectional processes like health selection. Unemployment has the same negative health effects in both boom and recession. Those in other labour market positions (studies, work) have impaired health during recession, but without long-term effects. Macrolevel structures of importance for how labour market position caused poor health, include class and gender structures and processes, as well as labour market policies which were beneficial for health.

**Fig. 1:**
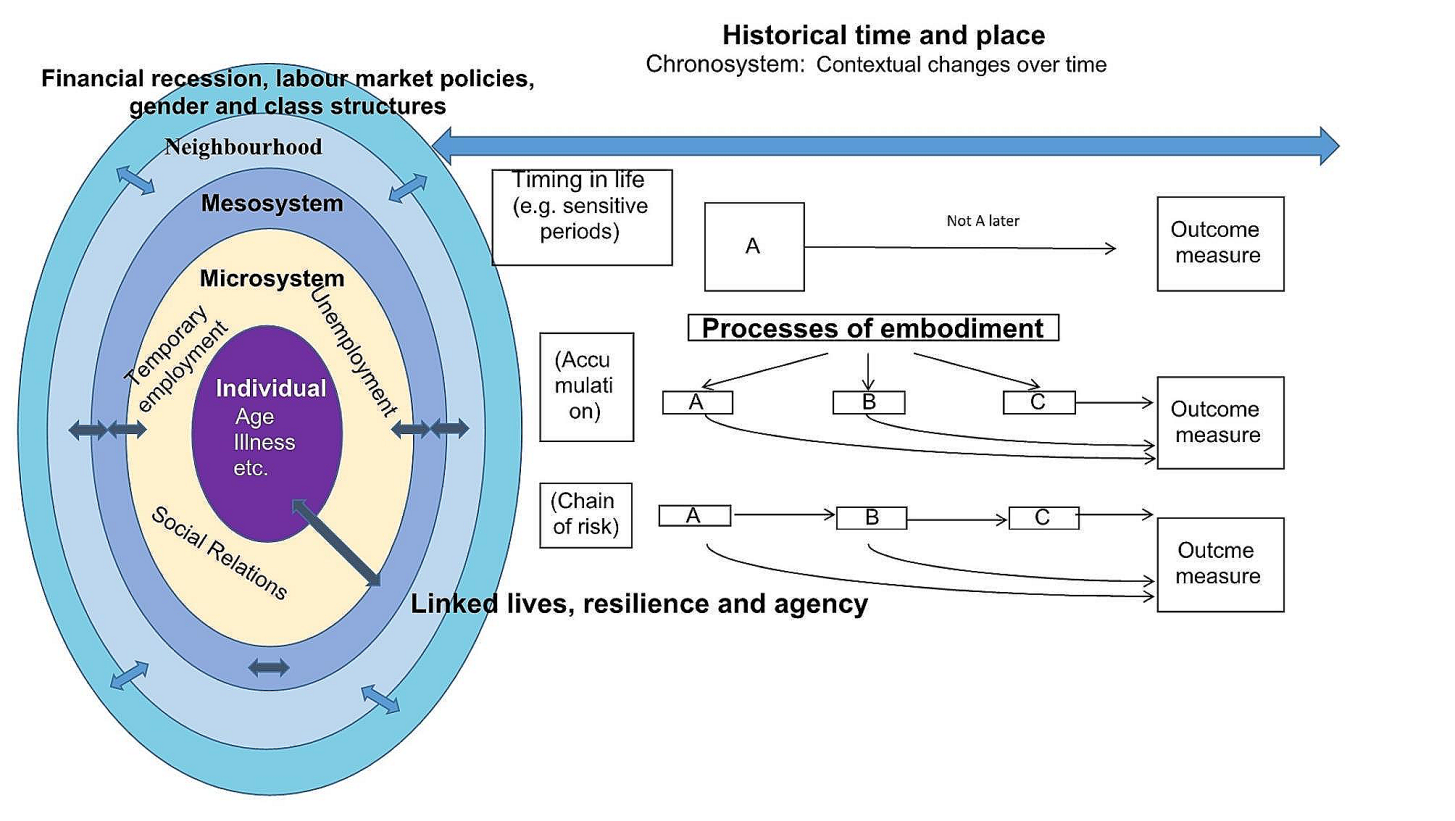
A contextual life-course model. Conceptualization of the Bronfenbrenner model on various societal levels and the models of life-course epidemiology in relation to our empirical findings in the integrative review

Our contextual life-course model, which integrates Bronfenbrenner’s Ecological Systems Theory model [3] with various temporal dimensions (see Fig. 1) is fruitful for providing a deeper understanding of how labour market conditions in various contexts influence mental health over life. As suggested by Elder [1], a mixed-method design, combining quantitative and qualitative methods, contributed to further development of the temporal dimensions and to new themes such as the impact of agency to improve mental health over life in dyadic relations at various microlevel settings.

According to Bronfenbrenner, research on the ecological system of human development should preferably include experiments involving the systems mentioned [3]. However, experiments performed in a laboratory lacking a minimum of context, proved to be a problematic approach heavily criticized by himself. Instead, he suggested that ‘natural experiments’ represented by implementation of changes in relevant policies, could be a fruitful approach if they could be operationalized in a clever mode. The huge and unexpected financial crisis of the early 1990s can be regarded as such a macrolevel experiment, which hit human beings differently depending on which developmental phase and which context they were in. This “experiment” is part of our metasynthesis. Other experiments, which are also part of our paper, consist of labour market policies and our analyses indicate their possible health promoting effects.

Our development of the contextual life-course model is of special importance to understand the health consequences of the rapidly developing labour market. Another framework for understanding the process of the gradual deterioration of the social rights components of the standard employment contract between employer and employee in a developed welfare state – i.e., full-time employment with income and employment security, paid vacation and pension rights, health insurance, functioning work safety measures and workplace democracy – has been proposed by Guy Standing [82].

Theoretical models are needed to further develop life-course epidemiology in relation to the rapidly changing labour market, as frameworks can help to structure ideas as well as explain disease distribution and causal relations between exposures and outcomes.

Our overall conclusion is that the labour market conditions surrounding the individual are of crucial importance for the embodiment of mental health over life, at the same time as individual agency can be health promoting. Mental health can be improved by societal efforts in regulations of the labour market.

## Data Availability

The datasets analysed during the current study are not publicly available due to the Swedish Act (SFS 2003:460) on ethical review of research involving humans, which does not permit sensitive data on humans to be freely shared. Parts of the datasets are available after ethical permission and after request to the PI (AH).
